# Primary ovarian small cell carcinoma of hypercalcemic type in a pregnant woman: A case report

**DOI:** 10.1097/MD.0000000000020387

**Published:** 2020-07-24

**Authors:** Min Feng, Kaixuan Yang, Lian Xu, Yan Zhang, Juan Zou

**Affiliations:** a Department of Pathology, West China Second University Hospital, Sichuan University; b Key Laboratory of Birth Defects and Related Diseases of Women and Children (Sichuan University), Ministry of Education, Renmin NanLu Chengdu, Sichuan, P.R. China.

**Keywords:** diagnosis, hypercalcemic type, ovarian small cell carcinoma, pregnancy

## Abstract

**Rationale:**

Ovarian small cell carcinoma of hypercalcemic type (OSCCHT) is a relatively rare and highly fatal gynecological malignancy of unknown histogenesis, affecting mainly girls and young women. OSCCHT occurring during pregnancy is an uncommon event, and preoperative diagnosis of this malignancy is much more difficult in pregnant than non-pregnant women. The aim of this study was to describe a rare case of primary OSCCHT in a pregnant woman and to review the current literature.

**Patient concerns:**

Here we present a case of OSCCHT in a 21-year-old patient in the 32nd week of gestation, who had abdominal pain and irregular vaginal bleeding for 5 hours. Because placental abruption, stillbirth, and hemorrhagic shock were suspected, she subsequently underwent diagnostic laparotomy. During the hysterotomy delivery and exploratory laparotomy, we found a dead fetus in the uterus and a large tumor mass arising from her left ovary. Plasma-based detection showed that the patient had a slightly elevated parathyroid hormone (PTH) level and normal serum calcium. After surgery, her serum PTH levels returned to normal.

**Diagnosis and interventions:**

The patient was initially treated with surgery. She underwent total abdominal hysterectomy with bilateral salpingo-oophorectomy, as well as the following additional procedures: appendectomy, sigmoidectomy, debulking of extra-ovarian tumor, lymph node dissection, and peritoneal biopsies. The patient, who was in the third trimester of pregnancy, was diagnosed with OSCCHT that was confirmed to be Stage III. She was recommended chemotherapy after surgery, but she declined chemotherapy.

**Outcomes:**

Unfortunately, the patient died 5 months after surgery.

**Lessons:**

OSCCHT is a very rare and highly aggressive tumor type. The clinical symptoms of this tumor are nonspecific, and pathological examination remains the gold standard for diagnosis. Most patients are diagnosed with advanced stage disease and do not respond to chemotherapy. The prognosis of OSCCHT is generally poor, and no treatment guidelines are available as yet. For pregnant woman, OSCCHT is especially harmful to the mother and may indirectly lead to the death of the fetus.

## 1 Introduction

Ovarian small cell carcinoma of hypercalcemic type (OSCCHT) is a very rare and highly aggressive tumor type, which usually occurs in young women.^[1,2]^ Although its treatment combines surgery, chemotherapy, and radiotherapy, this rare tumor is thought to be a highly fatal neoplasm with poor prognosis; overall survival is 1 to 2 years in most cases.^[2]^ This entity was first fully described by Dickersin et al in 1982.^[3]^ To this day, nearly 300 cases have been reported in the literature.^[1]^ OSCCHT has no specific clinical manifestations, mainly presenting as unilateral adnexal masses with equal incidence on the left and right side. Two-thirds of cases are associated with hypercalcemia, but only 10% of patients have symptoms of hypercalcemia.^[4,5]^ Due to its unclear familial history and vague symptoms, most patients with OSCCHT present late with advanced disease.^[1,5]^ There is little knowledge regarding patients diagnosed with OSCCHT during pregnancy, and only a limited number of case reports can be found in the literature.^[6]^ Here, we report the case of a young woman in the third trimester of pregnancy who was diagnosed with OSCCHT, to investigate the clinicopathological characteristics and diagnosis of this disease.

## 2 Case presentation

**Figure 1:**
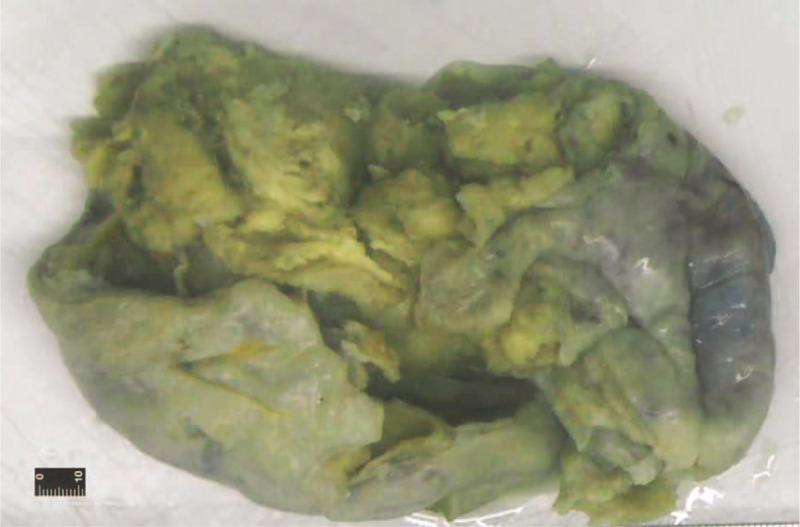
Photograph of the ovarian mass, after formalin fixation. The tumor had a soft consistency, an incomplete and thin pseudocapsule, and gray-to-yellowish cut surface, with significant necrosis and hemorrhage.

A 21-year-old G0P0 woman who had menopaused for 32 weeks was referred to our hospital with abdominal pain and irregular vaginal bleeding for 5 hours. Because no fetal heart sounds were detected upon examination, placental abruption, stillbirth, and hemorrhagic shock were suspected. The patient subsequently underwent cesarean delivery and exploratory laparotomy. Laboratory analyses showed that the CA125 level was 187 U/mL (normal range: <35.0 U/mL), the parathyroid hormone (PTH) level was 8.68 pmol/L (normal, 1.6–8.3 pmol/L), and serum calcium was normal. During diagnostic laparotomy, we found a dead fetus in the uterus, as well as a large solid cystic tumor arising from the left ovary, filling the true pelvis: the right ovary appeared normal on gross visual inspection. The tumor had a soft consistency, with rupturing small cystic cavities. The left ovarian mass was removed, and frozen sections were diagnosed as neoplasm, possible granulosa cell tumor, favoring small cell carcinoma. Complete surgical staging was performed, including hysterectomy, bilateral adnexectomy, pelvic and para-aortic lymph node dissection, appendectomy, partial sigmoid colon resection, omentectomy, and peritoneal biopsies. Grossly, the tumor measured 20.5 cm × 18.3 cm × 15.5 cm (Fig. 1; after formalin fixation); it had a soft consistency, an incomplete and thin pseudocapsule, and a gray-to-yellowish cut surface, with substantial necrosis and hemorrhage.

**Figure 2:**
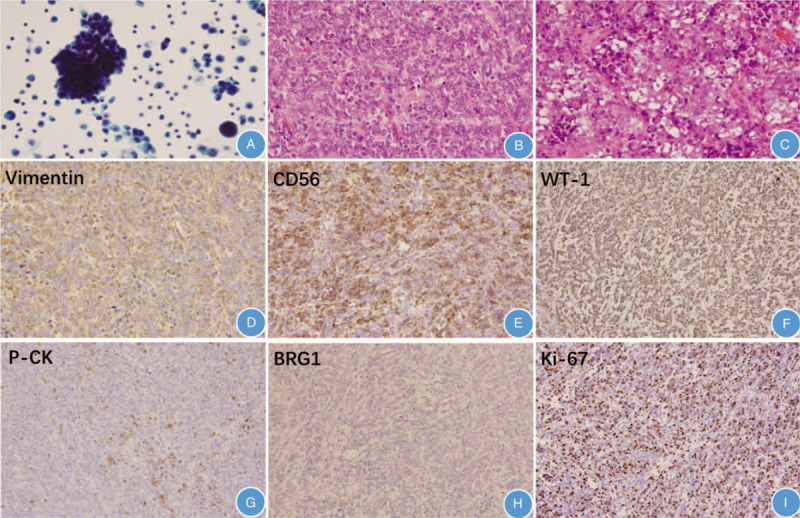
Hematoxylin and eosin (H&E) and immunohistochemical staining of ovarian small cell carcinoma of hypercalcemic type. (A) Cytological examination revealed many tumor cells in peritoneal washings. Cells were small and round, individually present or in loose clusters, with increased nucleoplasmic ratio, reduced cytoplasm, and deep chromatin staining (Papanicolaou stain, 400× magnification). (B) Diffuse solid pattern of small, round, monomorphic cells with numerous mitoses (H&E, 200×). (C) Tumor cells with abundant cytoplasm, clear nuclei, and nucleoli (H&E, 200). (D) Vimentin was diffusely positive (H&E, 200×). (E) CD56 was diffusely positive (H&E, 200×). (F) WT-1 was diffusely positive (H&E, 200×). (G) P-CK was focally positive (H&E, 200×). (H) BRG1 was negative (H&E, 200×). (I) The MKI-67 proliferative index was approximately 80% (H&E, 200×).

In peritoneal washings, we found many tumor cells upon cytological examination. The tumor cells were small and round, individually present or aggregated into loose clusters, with a considerably increased nucleoplasmic ratio, reduced cytoplasm volume, and deep chromatin staining (Fig. 2A). Histological examination showed a diffuse solid pattern of small, round, monomorphic cells with numerous mitoses (Fig. 2B). In some places, the tumor cells had abundant cytoplasm as well as clear nuclei and nucleoli (Fig. 2C). Immunohistochemical staining revealed that the tumor cells were diffusely positive for vimentin, WT-1, and CD56. Focal expression was found for EMA, P-CK, and CD99; CA125, ER, PR, Pax-8, TTF-1, SYN, CGA, calretinin, inhibin, CD30, OCT3/4, LCA, and BRG1 were not expressed (Fig. 2D–H). The MKI-67index was estimated to be approximately 80% (Fig. 2I). Finally, histopathological examination and immunohistochemistry (IHC) results of the patient's specimens confirmed the diagnosis of OSCCHT of the left ovary. In addition, the right ovary, bilateral fallopian tubes, uterus, omentum, sigmoid colon, and round ligament were all positive for disease; the appendix and pelvic cavity lymph nodes showed no evidence of the tumor. The patient was confirmed to have Stage III disease. Chemotherapy was recommended; however, she declined chemotherapy and died 5 months after surgery.

As this was a case report, ethical approval was not necessary. The patient's parents provided informed consent to collect data for publication.

## 3 Discussion

Primary ovarian small cell carcinoma (OSCC) is an aggressive gynecological malignancy with a tendency for early distant metastases and a low 5-year survival rate.^[1]^ There are 2 types of ovarian small cell carcinomas: hypercalcemic and pulmonary. In general, the incidence of epithelial ovarian cancer is increased in older patients (age >50 years), but OSCC of hypercalcemic type (OSCCHT) is a rare neoplasm that tends to affect young women, with an average age at diagnosis of 23 years.^[1]^ Diagnosis of OSCCHT during pregnancy is extremely rare.^[6]^ The clinical symptoms of OSCCHT are nonspecific: abdominal pain, abdominal distension, and vaginal bleeding.^[1,3]^ In addition to unclear and vague symptoms of OSCCHT, tumor markers that are normally useful in epithelial ovarian cancers provide little information in OSCCHT. Definitive preoperative diagnosis of OSCCHT is frequently difficult,^[7,8]^ especially in pregnant woman. Therefore, pathological examination remains the gold standard for the diagnosis of this disease.

It has been reported that two-thirds of patients with OSCCHT present with hypercalcemia.^[1,4]^ However, symptoms of hypercalcemia, for example, polyuria, polydipsia, anorexia, nausea, vomiting, constipation, muscle weakness, bone pain, decreased concentration, confusion, fatigue, and coma are reported in <10% of these patients.^[2]^ Related studies have reported variable serum calcium levels of 2.43 to 4.80 mmol/L, with an average of 3.68 mmol/L.^[9]^ The mechanism of hypercalcemia development associated with this tumor is not clearly understood, but it is generally believed that hypercalcemia is caused by tumor cells. Unlike the mechanism of neuroendocrine tumors, the mechanism in OSCCHT may be related to direct secretion by the tumor of PTH-related protein (PTHrP), as detected with IHC. In some cases, serum calcium levels return to normal after surgery, reflecting that serum calcium levels correspond with surgical treatment, which might be a useful means of monitoring this disease.^[10]^ Some studies recommend that CA125 could be a very useful tumor marker, which is elevated in approximately 75% of patients at diagnosis. However, tumor markers should be used with caution in the diagnosis of ovarian cancer during pregnancy.^[7]^ During normal pregnancy, especially in the first and last trimester, decidua and granulosa cells produce CA125, reducing its diagnostic value for ovarian cancer.^[8]^ In the present case, the 21-year-old patient was in the last trimester of pregnancy; she had a slightly elevated level of PTH and normal serum calcium levels at diagnosis. After surgery, PTH levels returned to normal, indicating that increased PTH levels may be caused by tumor cells. Similar to some previous reports, the tumor marker CA125 was mildly elevated at diagnosis in this young patient.

In OSCCTH, tumors are generally large in size, with an average diameter of 15 to 16 cm, and a nodular or lobulated appearance with mostly solid or partially cystic sections accompanied by visible hemorrhage and necrosis.^[1,5]^ Histologically, the growth pattern of OSCCTH is solid and trabecular, with typical follicle-like spaces and a sheet-like arrangement of small monomorphic cells, with scanty cytoplasm and round, ovoid, small nuclei containing single, small nucleoli.^[7,10]^ Mitotic figures and necrosis are frequent. In some rare cases, mucinous glands, mucinous signet-ring cells, spindle-cell sarcomatoid change, large cells, and rhabdoid cytomorphology have been described.^[4,9]^ Immunohistochemically, OSCCHT is positive for WT-1 and vimentin, with focal expression of cytokeratins (AE1/AE3 or EMA). Moreover, expression of p53, CD56, calretinin, CD10, α-smooth muscle actin, and TOP2A have been described.^[11]^ All IHC markers are nonspecific, and the diagnosis is made mainly on the basis of morphology. Recently, several authors have shown that OSCCHT is characterized by the inactivation of the *SMARCA4* gene (encoding the BRG1 protein), a member of the SWI/SNF chromatin-remodeling gene complex, which is also frequent in malignant rhabdoid tumors, resulting in a loss of BRG1 protein expression with IHC.^[12–15]^ Thus, the absence of SMARCA4/BRG1 immunostaining may prove very useful in the diagnosis of OSCCHT.^[12,15]^ In the present case, the tumor cells were negative for BRG1 protein, which further supported the final diagnosis of OSCCHT. A number of neoplasms can be confused with OSCCHT, including granulosa cell tumors, primary or secondary neuroendocrine tumors, lymphoma, primitive neuroectodermal tumor, Ewing sarcoma, dysgerminoma, melanoma, round cell sarcoma, and desmoplastic small cell tumors. Several non-neuroendocrine tumors, such as teratoma, sex-cord stromal tumors, and Sertoli-Leydig cell tumors, may show neuroendocrine differentiation. These tumors can usually be distinguished from OSCCHT by identifying non-neuroendocrine components.

Until now, the etiology of OSCCHT has remained obscure. Whereas authors who performed IHC analysis of OSCCHT have postulated a germ cell-derived tumor, other authors have discussed OSCCHT as an epithelial-like originating tumor, and genetic analysis of OSCCHT tumor specimens have identified an inhomogeneous tumor entity.^[14]^ As mentioned above, accumulating evidence has shown that *SMARCA4* is mutated in 75% to 100% of OSCCHT cases,^[11,13]^ which is a similar genetic profile to malignant rhabdoid tumors. Some authors have proposed renaming OSCCHT as “malignant rhabdoid tumor of the ovary,”^[12,13,16]^ although OSCCHT is classified as a miscellaneous ovarian tumor by the World Health Organization. This view is supported by the following features shared by both OSCCHT and malignant rhabdoid tumor: occasional familial occurrence, frequent hypercalcemia, and immunohistochemical polyphenotypia.^[12]^ Additionally, in vivo studies have revealed that the simultaneous presence of c-Met in 41% of OSCCHT-1 cells, reduced proliferative capacity, and decreased tumor size are observed after siRNA-mediated c-Met knockdown in OSCCHT-1 cells, demonstrating that in vivo inhibition of these pathways contributes to attenuation of OSCCHT tumor growth.^[17]^

To the best of our knowledge, although the occurrence of ovarian masses in pregnancy is relatively common, most are functional and resolve spontaneously within the first 16 weeks of pregnancy. Nevertheless, a small proportion of pregnant women (0.2%–2%) are diagnosed with ovarian cancer, mostly in the first trimester.^[18,19]^ Most ovarian cancers diagnosed during pregnancy are malignant germ cell tumors and epithelial cancers^[18]^; OSCCHT in pregnancy is extremely rare. Adnexal masses in premenopausal women are often found incidentally and are mostly of little clinical relevance. In pregnancy, making a proper diagnosis using imaging can be challenging because of the enlarged uterus and pregnancy-related morphologic changes in the ovaries. The 2 most commonly used imaging modalities to evaluate adnexal masses in pregnancy are ultrasonography (US) and magnetic resonance imaging (MRI) because these are safe, widely assessable, and have high sensitivity and specificity. But use of the MRI contrast agent gadolinium is discouraged as it crosses the placenta and is excreted into the amniotic fluid.^[20]^ A study by Bekiesińska-Figatowska et al^[21]^ of about 43 symptomatic pregnant women between 9 and 33 weeks’ gestation who required an imaging work-up suggested that MRI is the best imaging modality for pregnant women when US is inconclusive, when masses are too large to fully assess with US, or when there is a high risk of malignancy. Pregnancy affects growth invasion and metastasis in ovarian cancer, and ovarian cancer increases various maternal and fetal complications. Owing to a lack of regular abdominal US examinations during the pregnancy period, the adnexal mass was not found in the present patient and the mass grew larger within the enlarged uterus. Even more unfortunately, her fetus died in the uterus.

The tendency for rapid progression and high recurrence of OSCCHT makes treatment a challenge. Treatment of this disease is stage dependent and there is no standard treatment.^[5]^ In early stages, radical surgery is advised, usually followed by adjuvant chemotherapy with cisplatin and etoposide. For advanced-stage disease, multiple combinations of nearly all chemotherapy agents have been tried but with little success.^[22]^ Patients with recurrent disease may respond to salvage surgery, chemotherapy, and radiotherapy; some patients have been treated with high-dose chemotherapy and stem cell rescue.^[23,24]^ Despite various radical approaches, the 1-year survival rate in patients with OSCCHT is only around 50%. In a large series including 150 patients, 33% with stage IA disease were alive and disease-free at 5 years; however, only 10% with stage IC or stages II, III, and IV remained alive.^[4]^ It has been reported that indicators of good prognosis are localized tumor stage, size smaller than 10 cm, and patient age above 30 years. Tumors without a large contingent of cells are also associated with significantly higher survival.^[4]^ Recently, blocking of PD1/PD-L1 interaction is being studied in several malignancies, with good responses. Some data have suggested that OSCCHT are immunogenic tumors and exhibit biologically significant levels of T-cell infiltration and PD-L1 expression.^[25]^ A study by Yaghmour et al^[11]^ revealed that OSCCHT had high expression of PD1 and PD-L1; the therapeutic potential of this pathway needs to be explored. In clinical application, several patients with OSCCHT have received anti-PD1 immunotherapy and some benefit has been reported.^[25]^ More recently, a study described a successful case of advanced-stage OSCCHT of the left ovary treated with cytoreductive surgery, semi-intense chemotherapy, high-dose consolidative chemotherapy, autologous hematopoietic stem cell transplantation, and pelvic radiation, which resulted in good long-term survival.^[5]^

Many factors must be considered in patients with ovarian cancer during pregnancy, including the patient's desire for the pregnancy, stage of disease, and gestational age at diagnosis. Optimal treatment involves balancing the benefit of treatment for the mother while minimizing harm to the fetus. The outcome of patients with ovarian cancer diagnosed in pregnancy is similar to that of patients who are not pregnant, and stage of disease is the strongest independent prognostic factor for survival.^[18]^ However, several studies have showed that lactating patients diagnosed with ovarian cancer had a worse outcome than non-pregnant patients.^[18,20]^ Owing to the rarity of the tumor and a lack of large series evaluating therapeutic strategies, more case series reports and multicenter analyses are needed to further investigate this rare disease and optimize clinical treatments.

In summary, hypercalcemic small cell carcinoma of the ovary is a rare disease. A rapidly progressive and highly malignant tumor, OSCCHT complicating pregnancy is an extremely rare event, but it is highly suspected to cause problems in fetal development or even fetal death, as in this case. The final diagnosis mainly relies on clinical information, morphology, and IHC, The loss of SMARCA4/BRG1 expression is thought to be a useful diagnostic marker of OSCCHT. Management with multimodal therapy, including stem cell transplantation and immunotherapy, should be considered in the treatment of these devastating ovarian tumors. Awareness about the possibility of ovarian cancer in pregnancy is important, and careful evaluation of adnexal masses in pregnancy is required to avoid delay in diagnosis. We hope that with regular antenatal care and improved therapies, the survival time can be prolonged and the fetus may survive.

## Author contributions

**Acquisition of data**: Min Feng, Kuaixuan Yang, Yan Zhang.

**Analysis and interpretation of data**: Min Feng, Lian Xu, Juan Zou.

**Conceptualization:** Min Feng, Kaixuan Yang, Juan Zou.

**Critical revision of the manuscript for important intellectual content**: Kaixuan Yang, Juan Zou.

**Data curation:** Kaixuan Yang, Lian Xu, Yan Zhang.

**Drafting of the manuscript**: Min Feng.

**Formal analysis:** Min Feng, Lian Xu.

**Methodology:** Yan Zhang.

**Producing the figures:** Lian Xu.

**Study concept and design:** Min Feng.

**Visualization:** Kaixuan Yang, Juan Zou.

**Writing – original draft:** Min Feng.

## Correction

The grant information for Department of Science and Technology of Sichuan Province was corrected from 2018SZ0241 to 2018SZ0241 and 2017JY0254.
